# Transforming Growth Factor-β Signaling Regulates Tooth Root Dentinogenesis by Cooperation With Wnt Signaling

**DOI:** 10.3389/fcell.2021.687099

**Published:** 2021-06-29

**Authors:** Ran Zhang, Jingting Lin, Yang Liu, Shurong Yang, Qi He, Liang Zhu, Xiao Yang, Guan Yang

**Affiliations:** ^1^State Key Laboratory of Proteomics, Beijing Proteome Research Center, National Center for Protein Sciences (Beijing), Beijing Institute of Lifeomics, Beijing, China; ^2^Department of Oral Pathology, Peking University School and Hospital of Stomatology, Beijing, China; ^3^Department of Prosthodontics, Peking University School and Hospital of Stomatology, National Clinical Research Center for Oral Diseases, National Engineering Laboratory for Digital and Material Technology of Stomatology, Beijing Key Laboratory of Digital Stomatology, Beijing, China

**Keywords:** TGF-β type II receptor, *Wntless*, tooth root, odontoblast, dentin

## Abstract

Proper differentiation of odontoblasts is crucial for the development of tooth roots. Previous studies have reported the osteogenic/odontogenic potential of pre-odontoblasts during root odontoblast differentiation. However, the underlying molecular pathway that orchestrates these processes remains largely unclear. In this study, ablation of *transforming growth factor-*β *receptor type 2* (*Tgfbr2*) in root pre-odontoblasts resulted in abnormal formation of root osteodentin, which was associated with ectopic osteogenic differentiation of root odontoblasts. Disrupting TGF-β signaling caused upregulation of Wnt signaling characterized by increased *Wnt6*, *Wnt10a*, *Tcf-1*, and *Axin2* expression. Interestingly, inhibiting Wnt signaling by deleting *Wntless* (*wls*) in *Osteocalcin* (*Ocn*)*-Cre; Tgfbr2^*fl/fl*^; Wls^*fl/fl*^* mice or overexpressing the Wnt antagonist *Dkk1* in *Ocn-Cre; Tgfbr2^*fl/fl*^; ROSA26^*Dkk*1^* mice decreased ectopic osteogenic differentiation and arrested odontoblast differentiation. Our results suggest that TGF-β signaling acts with Wnt signaling to regulate root odontogenic differentiation.

## Introduction

The mechanism of crown dentinogenesis has been well studied; however, knowledge about root dentinogenesis is only emerging (Park et al., 2007; Zhang et al., 2015; Li et al., 2017). During root odontoblast differentiation, the cranial neural crest derived mesenchyme condenses around and continuously interacts with Hertwig’s epithelial root sheath (Huang et al., 2009). Subsequently, the apical papilla mesenchyme undergoes differentiation into pre-odontoblasts, which terminally differentiate to become odontoblasts (Ruch et al., 1995). This process is marked by the expression of several genes that encode collagenous and non-collagenous proteins that are also found in osteoblasts and the bone matrix (Gaikwad et al., 2001). The overlapping pattern of gene expression suggests the divergence of a common pool of progenitor cells for odontoblasts and osteoblasts. The molecular mechanism that controls the osteo/odontogenic differentiation of root dental mesenchyme remains largely unknown.

The results of our previous studies in mice revealed the critical function of Wnt/β-catenin signaling in root odontogenesis (Zhang et al., 2013); however, the precise regulatory role of Wnt signaling in root dentinogenesis remains to be clarified. Wnt proteins are a major family of developmentally important signaling molecules regulating multiple processes, including embryonic development, cell differentiation, and the specification of cell fate (Garcin and Habib, 2017; Ji et al., 2019; Xiong et al., 2019). Some Wnt ligands such as Wnt10a are spatiotemporally expressed with the differentiation stage of root odontoblasts during tooth development (Yamashiro et al., 2007). *Dkk1*, an inhibitor of Wnt signaling, is strongly expressed in pre-odontoblasts but its expression is lower in secretory odontoblasts (Fjeld et al., 2005). As a common Wnt target and a regulator of odontoblast differentiation, Runx2 induces transdifferentiation of odontoblasts into osteoblasts at the cell differentiation stage (Miyazaki et al., 2008). Therefore, Wnt signaling must be tightly controlled during root odontoblast differentiation; however, the molecular mechanisms that regulate this process remain unknown.

Transforming growth factor-β (TGF-β) signaling plays an important role in a broad range of cellular processes (Wu et al., 2016; Mullen and Wrana, 2017; Yu and Feng, 2019). Genetically modified mouse models have confirmed that a loss of responsiveness to TGF-β by odontoblasts results in aberrant pulp calcification; however, the underlying mechanisms have not been fully clarified (Wang et al., 2013; Ahn et al., 2015). Here, we specifically deleted *TGF-β receptor type 2* (*Tgfbr2*) from root pre-odontoblasts and observed reduced odontogenic differentiation but ectopic expression of *integrin bone sialoprotein* (*Ibsp*) in the root odontoblast layer with upregulated Wnt signaling. Inhibiting Wnt signaling by either deleting *Wntless* (*wls*) in *Ocn-Cre; Tgfbr2^fl/fl^; Wls^fl/fl^* mice or overexpressing *Dkk1* by breeding with floxed *ROSA26-loxP-stop-loxP-Dkk1* (*ROSA26^Dkk1^*) mice to generate *Ocn-Cre; Tgfbr2^fl/fl^; ROSA26^Dkk1^* mice not only decreased ectopic osteogenic differentiation, but also arrested odontoblast differentiation. Our data suggest that during early root odontoblast differentiation, TGF-β signaling might act with Wnt signaling to initiate odontogenesis; while during root odontoblast maturation, TGF-β signaling specified the odontogenic lineage by suppressing Wnt signaling.

## Materials and Methods

### Generation of Mouse Strain

Generation of *Osteocalcin* (*Ocn*, also known as *Bglap*)*-Cre; Tgfbr2^*fl/fl*^*, *Ocn-Cre; Wls^*fl/fl*^*, *Ocn-Cre; Tgfbr2^*fl/fl*^; Wls^*fl/fl*^*, and *Ocn-Cre; ROSA26^*EYFP*^* mice had been described previously (Yang et al., 2014). The conditionally *ROSA26^*Dkk*1^* mice (Wu et al., 2008) were bred with *Ocn-Cre; Tgfbr2^*fl/fl*^*. *Tgfbr2*^*fl/fl*^, *Tgfbr2^*fl/fl*^; Wls^*fl/fl*^*, *Tgfbr2^*fl/fl*^; ROSA26^*Dkk*1^* mice were used as controls. Mice were maintained on a C57BL/6 background and fed a normal rodent diet. For routine genotyping, *Cre* transgene were detected by PCR using primers described previously (Tan et al., 2007), and primers for *ROSA26^*Dkk*1^* locus were designed as follows: forward, 5′-TACGAAGTTATTAGGTCCCTCG-3′, and reverse, 5′-TTGTTCCCGCCCTCATAGA-3′. Experimental protocols were designed based on the recommendation of the Beijing Experimental Animal Regulation Board (SYXK/JING/2005/0031).

### Micro–Computed Tomography Analysis

The parameters of the Inveon MM system (Gantry-STD CT; Siemens) were set as follows: voltage of 60 kV, current of 220 mA, exposure time of 1500 ms, and effective pixel size of 8.89 μm. Sagittal images of the mandibles from postnatal day (P)30 *Tgfbr2^*fl/fl*^; Wls^*fl/fl*^* mice, *Ocn-Cre; Tgfbr2^*fl/fl*^* mice, *Ocn-Cre; Wls^*fl/fl*^* mice and *Ocn-Cre; Tgfbr2^*fl/fl*^; Wls^*fl/fl*^* mice (*n* = 4 mice/genotype) were captured with Inveon software. The slices through the apical foramen of the first mandibular molar were chosen for quantitative analysis.

### Histology and Immunohistochemistry

Mouse mandibles (*n* = 4 mice/genotype at P20, P30, and P120) were sectioned and were fixed in 4% paraformaldehyde/phosphate-buffered saline (PBS) overnight, then were decalcified and embedded in paraffin, and sectioned at 6 μm. Histological structures of mouse mandibular first molars were visualized using hematoxylin-eosin staining protocol. For immunohistochemical staining, antigen retrieval was performed in citrate buffer (pH 6.0) using a pressure cooker (Biocare Medical, Concord/California, biocare.net, DC2008). The primary antibodies used in the immunohistochemistry analyses were Tgfbr2 (Santa Cruz, sc-17792), β-catenin (BD Transduction Laboratories, 610153). Bound antibodies were visualized with diaminobenzidine, and sections were counterstained with hematoxylin, and observed under microscopy (Nikon, Eclipse E600).

### *In situ* Hybridization and Multicolor Chromogenic *in situ* Hybridization and Immunohistochemistry

Sectioning of mouse mandibles (*n* = 4 mice/genotype at P12 and P20) was performed using standard protocols. Briefly, mouse mandibles were fixed in 4% paraformaldehyde, embedded in paraffin, and sectioned at 6 μm. Sections were heated at 63°C, de-waxed in xylene, rehydrated through a graded series of alcohol washes, and post-fixed with 4% paraformaldehyde in 0.01 mol L^–1^ PBS. ^35^S-dUTP (Perkin-Elmer, NEG009T001MC) and digoxigenin-11-UTP (Roche Applied Science, 11277073910) were employed to label RNA probes for collagen type I, alpha 1 (Col1a1), Ocn, dentin sialophosphoprotein (*Dspp*) and Ibsp using the MAXIscript in vitro transcription kit (Ambion, T3/AM1316, T7/AM1312). In situ hybridization of paraffin sections using 35S-UTP-labeled probes was performed with standard procedures (Yang et al., 2014). Sections were immersed in K5 emulsion (Ilford, 02746-50) for 5–30 days before development. For *in situ* hybridization with digoxigenin-11-UTP labeled RNA probes, sections were incubated in 1:2000 AP-conjugated, polyclonal sheep anti-digoxigenin antibody (Roche Applied Science, 11093274910) at 4°C for 10 hours. Immunoreactive cells were visualized with HighDef red (AP) (Enzo Life Sciences, ADI-950-140-0030). The primary antibodies used for immunohistochemistry following in situ hybridization were monoclonal rabbit anti-green fluorescent protein (GFP) (Cell Signaling Technology, 2956). HRP-conjugated secondary antibodies were then applied (ZsBio, PV-6001). Immunoreactive cells were visualized with HighDef blue (HRP) (Enzo Life Sciences, ADI-950-151-0006). The nucleus were counterstained with methyl green, and observed under microscopy (Nikon, Eclipse E600).

### Statistical Analysis

Results (mean ± SD) were analyzed with Student’s *t*-test for independent samples or 1-way analysis of variance followed by Tukey’s *post hoc* test for pairwise comparisons (GraphPad Prism 7; GraphPad Software). Values of *P* < 0.05 were considered statistically significant.

## Results

### Disruption of *Tgfbr2* in Odontoblasts Leads to Abnormal Osteodentin Formation in the Root

To test the role of TGF-β signaling in root dentinogenesis during the development of tooth roots, we employed the *Ocn-Cre* strain to disrupt TGF-β signaling in *Ocn-Cre*; *Tgfbr2*^*fl/fl*^ mice. According to our previous study, *Ocn-Cre* recombinase activity is first detected in the primordial root at P10, in contrast to the intense expression in the developing crown odontoblasts and is active in the odontoblast lineages during root formation (Gao et al., 2009; Bae et al., 2013; Kim et al., 2013; Zhang et al., 2013). We then introduced *Ocn-Cre* and floxed *ROSA26-loxP-stop-loxP-EYFP* (*ROSA26*^*EYFP*^) mice to characterize which cell compartments in the tooth root harbored *Ocn-Cre-*mediated gene ablation. *Cre*-mediated recombination within the reporter locus activates enhanced yellow fluorescent protein (EYFP) expression and thus marks the recombined cells. *In situ* hybridization and immunohistochemical double labeling showed that odontoblasts lining the dentin layer are all EYFP^+^, but none of the EYFP^+^ cells in contact with Hertwig’s epithelial root sheath co-expressed *Col1a1*, *Ocn*, or *Dspp*,, which are markers of odontoblast differentiation (Supplementary Figure 1). These data demonstrate that the *Ocn-Cre* expression starts in pre-odontoblasts and leads to gene excision throughout the odontoblastic lineage.

**FIGURE 1:**
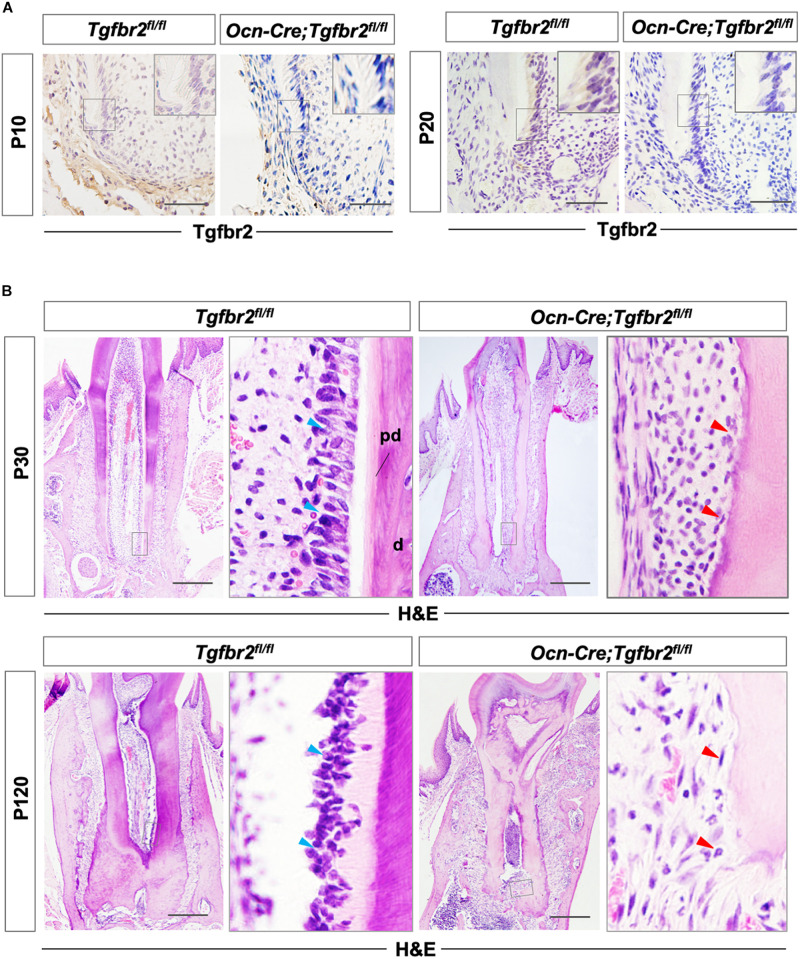
Disrupted tooth root development and formation of osteodentin in *Ocn-Cre; Tgfbr2^*fl/fl*^* mice. **(A)** Tgfbr2 is ablated in root odontoblasts in *Ocn-Cre*; *Tgfbr2*^*fl/fl*^ mice at P10 and P20 (*n* = 4 mice/per genotype). The top right panels show high-magnification image of the area boxed in gray on the left. **(B)** Abnormal root odontoblasts and root dentin formation in *Ocn-Cre*; *Tgfbr2*^*fl/fl*^ mice at P30 and P120. The right panels show the high-magnification image of the area boxed in gray on the left. Blue arrowheads indicate columnar-shaped odontoblasts in *Tgfbr2*^*fl/fl*^ mice, red arrowheads indicate irregular-shaped odontoblasts and formation of osteodentin in *Ocn-Cre*; *Tgfbr2*^*fl/fl*^ mice. pd, pre-dentin; d, dentin. Scale bars: 20 μm **(A)**, 100 μm **(B)**.

Tgfbr2 protein was detected in the membrane and cytoplasm of pre-odontoblasts at P10 and P20 and was successfully ablated in pre-odontoblasts and odontoblasts in *Ocn-Cre*; *Tgfbr2*^*fl/fl*^ mice as shown by immunohistochemistry (Figure 1A). The differentiating odontoblasts in *Tgfbr2*^*fl/fl*^ mice were columnar at P30 when development of the first molar roots was near completion with the nucleus aligned away from the newly formed dentin (Figure 1B, blue arrowheads). The root dentin was deposited in a well-organized structure with a lightly stained and demarcated layer of pre-dentin (Figure 1B). However, the cells lining the *Ocn-Cre*; *Tgfbr2*^*fl/fl*^ mouse tooth root were cuboidal and were entrapped in the newly formed matrix; this is similar to the way that osteoblasts differentiate (Figure 1B, red arrowheads). The pre-dentin layer of the mutant mice was lost and was replaced by osteodentin, a type of atubular dentin that resembles bone (Figure 1B).

### The Formation of Osteodentin Is Associated With Reduced Odontogenic Differentiation and Ectopic Osteogenic Differentiation

**FIGURE 2:**
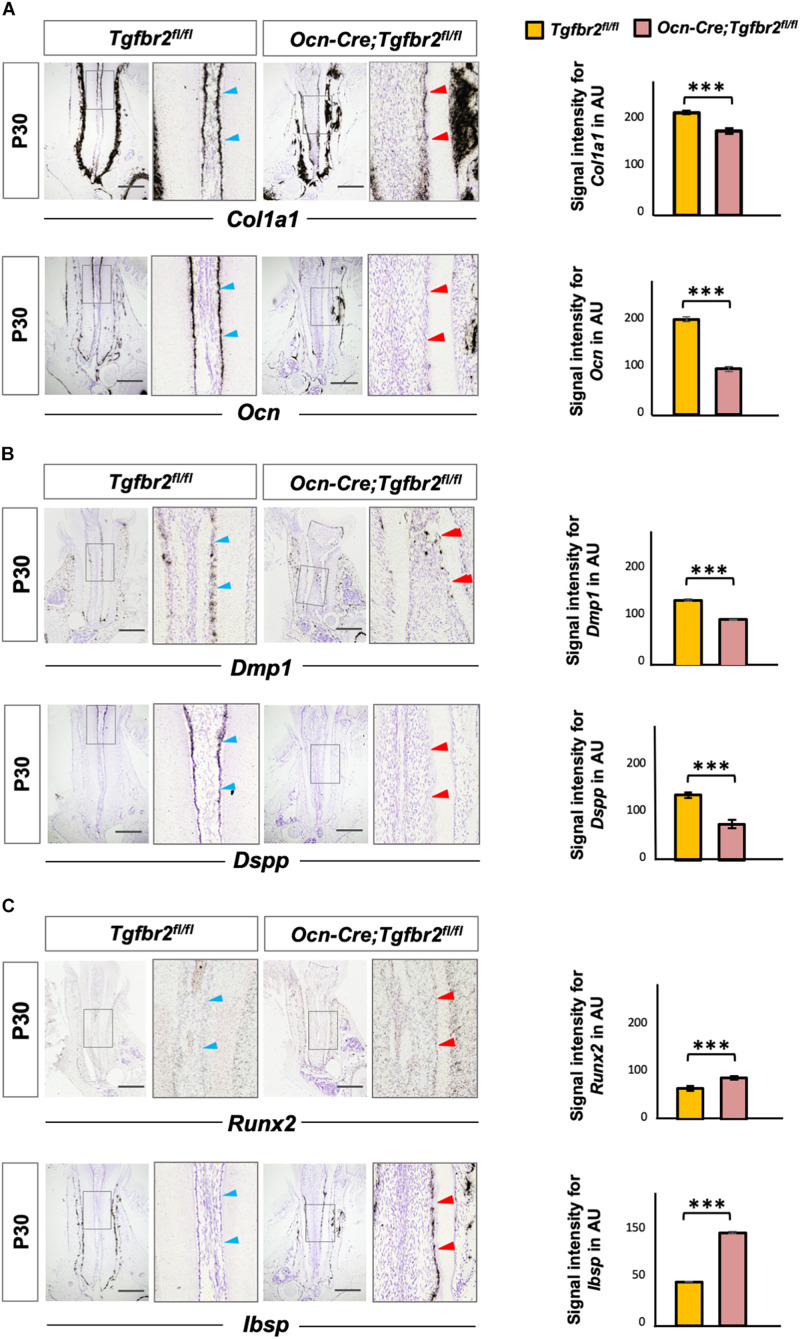
Decreased expression of odontogenic markers and ectopic expression of osteogenic markers in *Ocn-Cre*; *Tgfbr2*^*fl/fl*^ mice. **(A)** Decreased expression of *Col1a1* and *Ocn* in the root odontoblasts of *Ocn-Cre*; *Tgfbr2*^*fl/fl*^ mice were indicated by red arrowheads, as compared to that in the *Tgfbr2*^*fl/fl*^ littermates at P30 (blue arrowheads). **(B)** Reduced expression of *Dmp1* and diminished expression of *Dspp* in the root odontoblasts of *Ocn-Cre*; *Tgfbr2*^*fl/fl*^ mice at P30, red arrowheads indicate. **(C)** Upregulated expression of *Runx2*, and ectopic expression of *Ibsp* in the root odontoblasts of *Ocn-Cre*; *Tgfbr2*^*fl/fl*^ mice at P30, red arrowheads indicate. The right panels show the high-magnification image of the area boxed in gray on the left in **(A–C)**. The right images of bar graphs are quantification of signal intensity for *Col1a1*, *Ocn*, *Dmp1*, *Dspp*, *Runx2* and *Ibsp* expression as shown in **(A–C)**, respectively, using ImageJ. Data are presented as the means ± standard deviation (SD). ***P < 0.001, *n* = 4 areas from three mice. Blue arrowheads indicate expression of differentiation markers in *Tgfbr2*^*fl/fl*^ mice, red arrowheads indicate abnormal expression of differentiation markers in *Ocn-Cre*; *Tgfbr2*^*fl/fl*^ mice. AU, arbitrary unit. Scale bars: 100 μm.

The ectopic formation of osteodentin in *Ocn-Cre*; *Tgfbr2*^*fl/fl*^ mice suggests the possibility of a change in cell fate in mutant odontoblasts. To test this hypothesis, we performed *in situ* hybridization using osteo/odontogenic differentiation markers. The expression levels of *Col1a1* and *Ocn* decreased in the *Ocn-Cre*; *Tgfbr2*^*fl/fl*^ mice compared to *Tgfbr2*^*fl/fl*^ mice (Figure 2A, blue and red arrowheads). Expression of Dentin matrix protein 1 (*Dmp1*), a marker for early odontoblast differentiation, was evident in the root odontoblast layer of *Tgfbr2*^*fl/fl*^ mice, whereas it was sharply reduced in *Ocn-Cre*; *Tgfbr2*^*fl/fl*^ mice (Figure 2B, blue and red arrowheads). As a specific marker for mature odontoblasts, expression of *Dspp* was barely seen in the *Ocn-Cre*; *Tgfbr2*^*fl/fl*^ mice (Figure 2B, blue and red arrowheads). Interestingly, expression of the transcription factor *Runx2*, which is critical for osteogenic differentiation (Komori et al., 1997), increased in the odontoblast layer (Figure 2C, blue and red arrowheads). Strong ectopic expression of *Ibsp*, a marker for osteoblasts, was detected in the odontoblast layer of *Ocn-Cre*; *Tgfbr2*^*fl/fl*^ mice, whereas it was undetectable in *Tgfbr2*^*fl/fl*^ mice (Figure 2C, blue and red arrowheads). The ectopic expression of osteogenic differentiation markers suggested a change in cell fate in odontoblasts from *Ocn-Cre*; *Tgfbr2*^*fl/fl*^ mice.

### Increased Wnt Signaling Activity in Teeth From *Ocn-Cre*; *Tgfbr2*^*fl/fl*^ Mice

**FIGURE 3:**
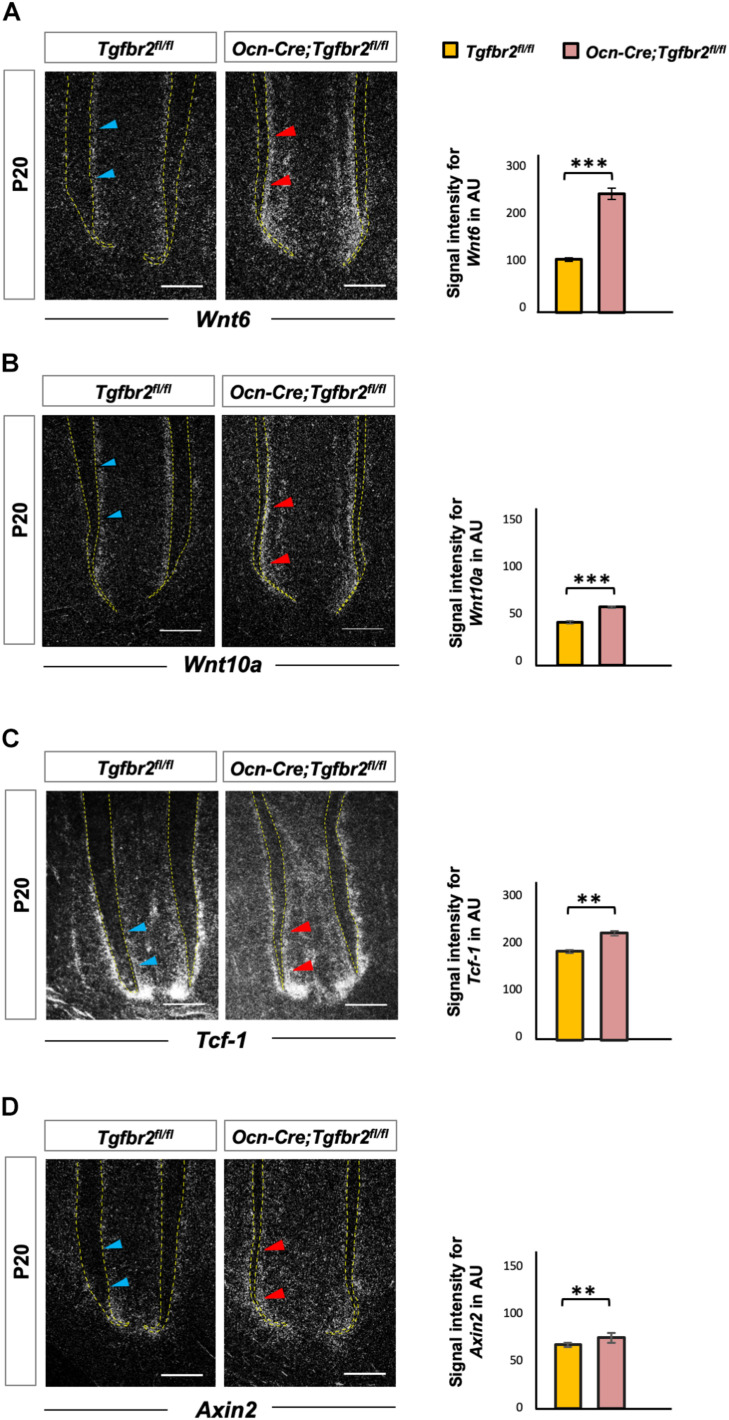
Increased Wnt signaling in the root pre-odontoblasts of *Ocn-Cre*; *Tgfbr2*^*fl/fl*^ mice. **(A,B)** Elevated expression of Wnt ligands, *Wnt6* and *Wnt10a*, in root odontoblasts of the *Ocn-Cre*; *Tgfbr2*^*fl/fl*^ mice at P20 as indicated by red arrowheads. **(C,D)** Increased expression of Wnt signaling read-out genes in the root odontoblasts of *Ocn-Cre*; *Tgfbr2*^*fl/fl*^ mice compared to the *Tgfbr2*^*fl/fl*^ littermates as indicated by red arrowheads at P20. *N* = 4 mice/per genotype in **(A–D)**. The right images of bar graphs are quantification of signal intensity for *Wnt6*, *Wnt10a*, *Tcf-1*, and *Axin2* expression as shown in **(A–D)** using ImageJ. Data are presented as the means ± standard deviation (SD). ***P < 0.001, **P < 0.01, *n* = 4 areas from three mice. Blue arrowheads in **(A–D)** indicate expression of Wnt ligands and related genes in *Tgfbr2*^*fl/fl*^ mice. Scale bars: 50 μm.

Wnt signaling is a key determinant of cell fate and a promotor of osteogenic differentiation in multiple cell types (Brack et al., 2007; Cawthorn et al., 2012; Zhu et al., 2019). We hypothesized that ectopic expression of *Ibsp* in mutant odontoblasts was due to over-activation of Wnt signaling. As a member of the canonical Wnt pathway, overexpressing Wnt6 could enhance the osteogenic differentiation of human dental papilla cells (hDPCs) with up-regulated expression of mineralization-related genes such as alkaline phosphatase (*ALP*), *Col1a1*, *Ocn*, *Osteopontin*, and *Ibsp* (Wang et al., 2010). Wnt10a has been reported to regulate osteogenesis in mesenchymal stem cells, and T cell factor 1 (*Tcf-1*), which is a nuclear transcription factor, potentially bound to the promoter region of *Runx2* and determined the osteogenic lineage in stem cells from human apical papilla cells (Zhou et al., 2019). Therefore, we detected expression of *Wnt6*, *Wnt10a*, and *Tcf-1* by *in situ* hybridization. Transcription of *Wnt6*, *Wnt10a*, and *Tcf-1* was upregulated in *Ocn-Cre*; *Tgfbr2*^*fl/fl*^ mice (Figures 3A–C, blue and red arrowheads). We also analyzed the expression of *Axin2*, a canonical Wnt target gene, and found it was increased significantly in *Ocn-Cre*; *Tgfbr2*^*fl/fl*^ mice (Figure 3D, blue and red arrowheads).

### Inhibition of Wnt Signaling in *Tgfbr2*-Deficient Mice Decreases Ectopic Osteogenic Differentiation and Arrests Odontoblast Differentiation

**FIGURE 4:**
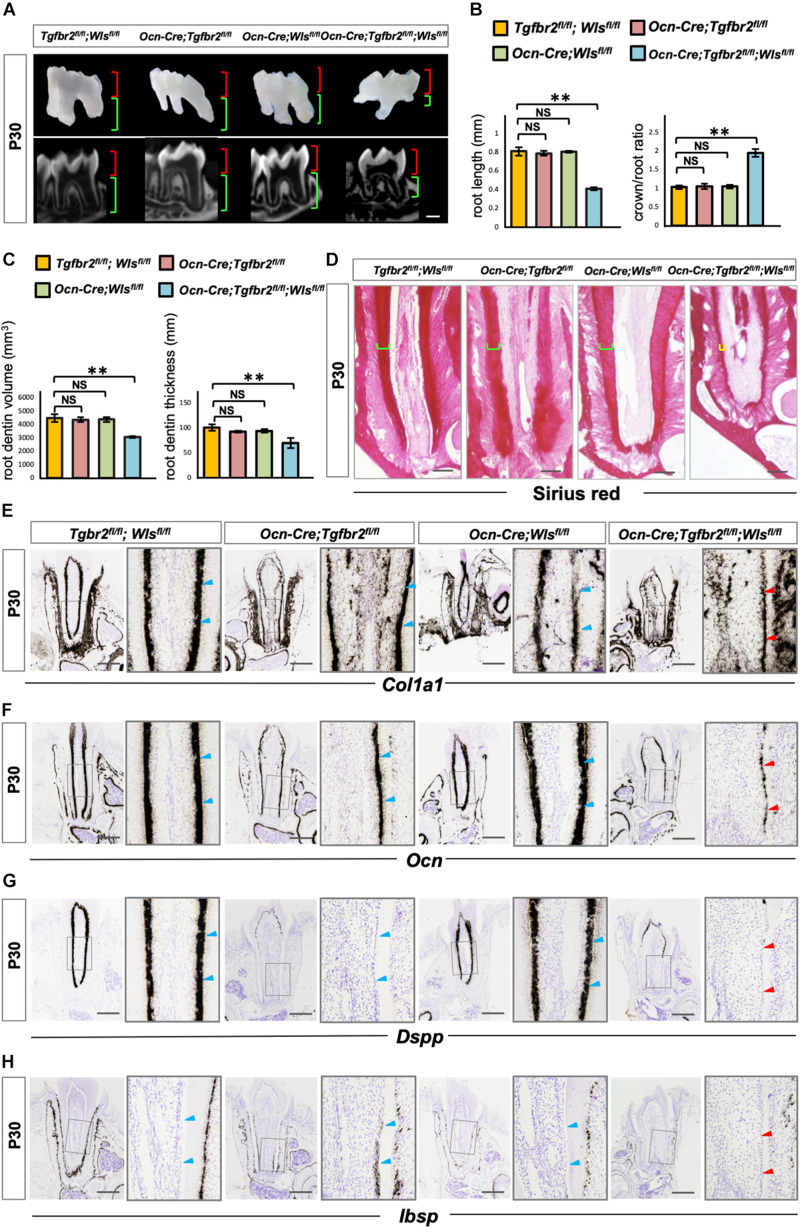
Arrested root odontoblast differentiation in *Ocn-Cre*; *Tgfbr2*^*fl/fl*^; *Wls*^*fl/fl*^ mice. **(A,B)** Significant short molar roots in *Ocn-Cre*; *Tgfbr2*^*fl/fl*^; *Wls*^*fl/fl*^ mice compared to *Tgfbr2*^*fl/fl*^; *Wls*^*fl/fl*^ mice, *Ocn-Cre*; *Tgfbr2*^*fl/fl*^ mice and *Ocn-Cre*; *Wls*^*fl/fl*^ mice at P30. Data are presented as the means ± standard deviation (SD). **P < 0.01, *n* = 4 mice/per genotype. **(C,D)** Reductions in root dentin volume and dentin thickness in *Ocn-Cre*; *Tgfbr2*^*fl/fl*^; *Wls*^*fl/fl*^ mice at P30. Data are presented as the means ± standard deviation (SD). **P < 0.01, *n* = 4 mice/per genotype. **(E,F)** Expression of osteo/odontogenic differentiation markers is sharply reduced in *Ocn-Cre*; *Tgfbr2*^*fl/fl*^; *Wls*^*fl/fl*^ mice at P30 (*n* = 4 mice/per genotype). **(G,H)** Diminished expression of *Dspp* and *Ibsp* in root odontoblasts of *Ocn-Cre*; *Tgfbr2*^*fl/fl*^; *Wls*^*fl/fl*^ mice at P30 (*n* = 4 mice/per genotype). Blue arrowheads indicate expression of differentiation markers in *Tgfbr2*^*fl/fl*^; *Wls*^*fl/fl*^ mice, *Ocn-Cre*; *Tgfbr2*^*fl/fl*^ mice and *Ocn-Cre*; *Wls*^*fl/fl*^ mice, red arrowheads indicate sharp reduction in expression of differentiation markers in *Ocn-Cre*; *Tgfbr2*^*fl/fl*^; *Wls*^*fl/fl*^ mice. The right panels show the high-magnification image of the area boxed in gray on the left in **(E–H)**. NS, non-significant. Scale bars: 300 μm **(A)**, 50 μm **(D)**, 100 μm **(E–H)**.

To further verify the involvement of upregulated Wnt signaling in the determination of osteo/odontogenic cell fate in *Ocn-Cre*; *Tgfbr2*^*fl/fl*^ mice, we specifically inhibited Wnt signaling by depleting *Wls*, an essential Wnt ligand transporter, to generate *Ocn-Cre*; *Tgfbr2*^*fl/fl*^; *Wls*^*fl/fl*^ mice. Tooth length was strikingly short in the *Ocn-Cre*; *Tgfbr2^*fl/fl*^; Wls^*fl/fl*^* mice (Figures 4A,B). Quantification of micro-computed tomography data and Sirius Red staining revealed a striking reduction in dentin volume and dentin thickness in *Ocn-Cre; Tgfbr2^*fl/fl*^; Wls^*fl/fl*^* mice compared to *Tgfbr2^*fl/fl*^; Wls^*fl/fl*^* mice, *Ocn-Cre; Tgfbr2^*fl/fl*^* mice, and *Ocn-Cre; Wls^*fl/fl*^* mice, respectively (Figures 4C,D). *Dkk1*, a secreted protein that functions as a negative regulator of Wnt signaling by forming a complex with LRP 5/6 to inhibit binding of Wnt ligands to receptors (Glinka et al., 1998; Wu et al., 2008), was introduced and overexpressed in pre-odontoblasts by Ocn-Cre and ROSA26Dkk1 mice. Similarly, the formation of root dentin in *Ocn-Cre; ROSA26^*Dkk*1^* mice was almost the same as that in *Tgfbr2^*fl/fl*^; ROSA26^*Dkk*1^* mice (Supplementary Figure 2B). In contrast, the roots were much shorter in the *Ocn-Cre; Tgfbr2^*fl/fl*^; ROSA26^*Dkk*1^* mice, and the dentin were thinner than that in the control littermates (Supplementary Figure 2B). Expression of the early differentiation markers *Col1a1* and *Ocn* decreased sharply in *Ocn-Cre; Tgfbr2^*fl/fl*^; Wls^*fl/fl*^* mice (Figures 4E,F, blue and red arrowheads), indicating a decreased differentiation ability at the early stage of odontoblast differentiation. As a major marker of odontoblastic differentiation, *Dspp* was undetectable in the root odontoblast layer of *Ocn-Cre; Tgfbr2^*fl/fl*^; Wls^*fl/fl*^* mice (Figure 4G, blue and red arrowheads). We did not observe expression of *Ibsp* in the dentin region of *Ocn-Cre; Tgfbr2^*fl/fl*^; Wls^*fl/fl*^* mice, which was evident in the *Ocn-Cre; Tgfbr2^*fl/fl*^* mice (Figure 4H, blue and red arrowheads). These data suggest that disrupting Wnt signaling in *Ocn-Cre; Tgfbr2^*fl/fl*^* mice led to arrested differentiation of root odontoblasts.

### β-Catenin Expression is Reduced in Differentiated Root Odontoblasts From *Ocn-Cre*; *Tgfbr2*^*fl/fl*^; *Wls*^*fl/fl*^ Mice

**FIGURE 5:**
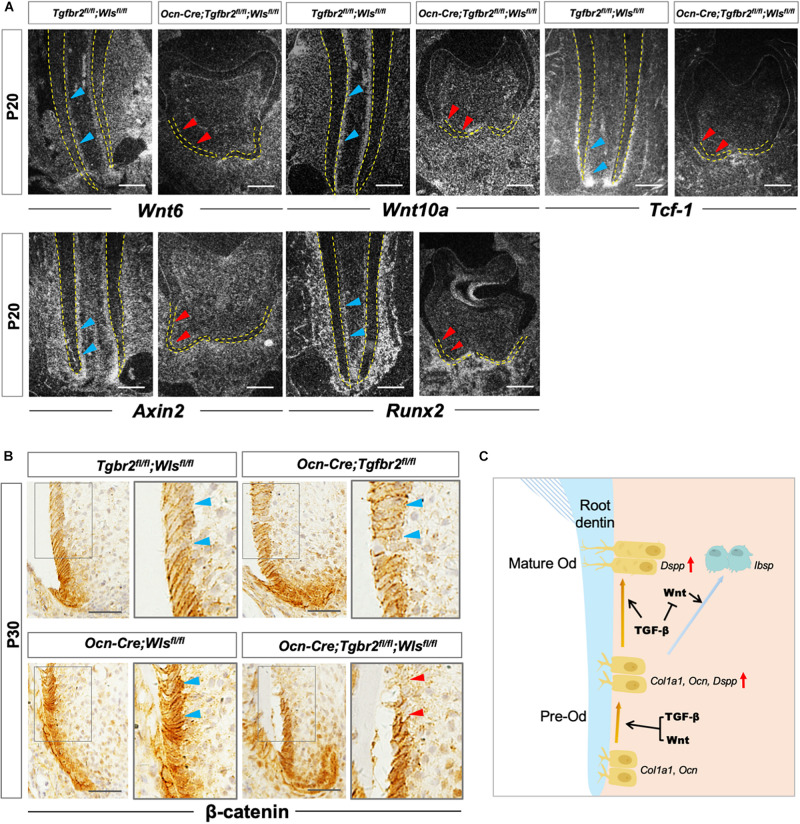
Compromised Wnt/β-catenin signaling in *Ocn-Cre; Tgfbr2^*fl/fl*^*; *Wls*^*fl/fl*^ mice. **(A)** Downregulated expression of Wnt related genes and *Runx2* in *Ocn-Cre*; *Tgfbr2*^*fl/fl*^; *Wls*^*fl/fl*^ mice indicated by red arrowheads at P20. **(B)** Reduced expression of β-catenin in odontoblasts of *Ocn-Cre*; *Tgfbr2*^*fl/fl*^; *Wls*^*fl/fl*^ mice indicated by red arrowheads at P30. **(C)** The scheme representation of possible mechanisms of TGF-β and Wnt signaling pathways in pre-odontoblast differentiation during tooth root development. Blue arrowheads indicate expression of *Wnt6*, *Wnt10a*, *Tcf-1*, *Axin2*, and *Runx2* in *Tgfbr2*^*fl/fl*^; *Wls*^*fl/fl*^ mice **(A)**, and expression of β-catenin in *Tgfbr2*^*fl/fl*^; *Wls*^*fl/fl*^ mice, *Ocn-Cre*; *Tgfbr2*^*fl/fl*^ mice, and *Ocn-Cre*; *Wls*^*fl/fl*^ mice **(B)**. Red arrowheads indicate downregulated expression of *Wnt6*, *Wnt10a*, *Tcf-1*, *Axin2*, and *Runx2* **(A)**, and reduced β-catenin expression in *Ocn-Cre*; *Tgfbr2*^*fl/fl*^; *Wls*^*fl/fl*^ mice **(B)**. *N* = 4 mice/per genotype in **(A,B)**. The right panels show the high-magnification image of the area boxed in gray on the left. Od, odontoblast. Scale bars: 50 μm **(A)** and 25 μm **(B)**.

Our previous study reported the critical role of β-catenin in the differentiation of root odontoblasts by showing the lack of expression of the odontogenic differentiation markers *Col1a1*, *Ocn*, and *Dspp* in *Ocn-Cre; Ctnnb1^*fl/fl*^* mice (Zhang et al., 2013). Similar to the previous study, the expression of odontoblastic differentiation markers in *Ocn-Cre; Tgfbr2^*fl/fl*^; Wls^*fl/fl*^* mice decreased significantly. We hypothesized that arrested root odontoblast differentiation was correlated to the reduced expression of β-catenin. We observed attenuated expression of *Wnt6*, *Wnt10a*, *Tcf-1*, and *Axin2* in root odontoblasts of *Ocn-Cre; Tgfbr2^*fl/fl*^; Wls^*fl/fl*^* mice by *in situ* hybridization (Figure 5A, red arrowheads). As the direct downstream target, *Runx2* expression was also downregulated in *Ocn-Cre; Tgfbr2^*fl/fl*^; Wls^*fl/fl*^* mice (Figure 5A, red arrowheads). Notably, β-catenin expression was reduced in the differentiated odontoblast layer of *Ocn-Cre; Tgfbr2^*fl/fl*^; Wls^*fl/fl*^* mice (Figure 5B, blue and red arrowheads), indicating that the integrity of β-catenin-mediated pathway might be required for TGF-β/Wnt signaling to initiate the root odontogenic differentiation.

## Discussion

The role of TGF-β signaling pathway that regulates odontoblast differentiation has been reported in several animal models (Oka et al., 2007*;* Kim et al., 2015). For example, Smad4-mediated TGF-β/bone morphogenetic protein signaling maintain Wnt activity in the dental mesenchyme to ensure a proper cranial neural crest cell fate decision during the early stage of tooth morphogenesis (Li et al., 2011). However, root odontoblast differentiation occurs at the late stage of tooth development and was thought to have a different regulatory mechanism from crown odontoblast differentiation. Indeed, during tooth root development, p-Smad2/3 is strongly expressed in dental papilla, moderately expressed in odontoblasts, indicating a stage-specific role of TGF-β signaling in root odontoblast differentiation (Li and Pan, 2018). Interestingly, many Wnt family members are also differentially expressed in the developing tooth root pre-odontoblasts and mature odontoblasts, for example, *Wnt6*, *Wnt10a* (Yamashiro et al., 2007), and their downstream targets *Tcf-1* and *Axin2*, suggesting that Wnt signaling might function in a synergic way with TGF-β signaling (Lohi et al., 2010). To clarify this, we generated a serial of *Ocn-Cre; Tgfbr2^*fl/fl*^*, *Ocn-Cre; Tgfbr2^*fl/fl*^; Wls^*fl/fl*^*, and *Ocn-Cre; Tgfbr2^*fl/fl*^; ROSA26^*Dkk*1^* mice, and provided *in vivo* evidences that TGF-β signaling is required for the root odontogenesis by cooperation with Wnt signaling, whereas inhibits osteogenic potential to insure the specified odontogenic lineage, which might be achieved through suppressing Wnt expression (Figure 5C). In accordance with our results, previous studies have also observed a calcified mass in the pulp chamber after *Tgfbr2* ablation in the dental mesenchyme, however, the cell source as well as the downstream effectors that produced the ectopic bone-like structures have not been demonstrated (Wang et al., 2013*;* Ahn et al., 2015). Study showed that overexpressing *Runx2*, a master regulator of osteoblast differentiation, in odontoblasts inhibits the terminal odontogenic differentiation and reversed them to a osteogenic phenotype (Miyazaki et al., 2008). Since Wnt signaling is an evolutionarily conserved intracellular signaling that plays an important role in the osteogenic differentiation, it is likely that TGF-β might inhibit Wnt signaling to reduce *Runx2* expression, which might explain how TGF-β signaling functions in directing the osteo/odontogenic phenotype of mature root odontoblasts. However, the molecular mechanisms underlying these regulations need to be further clarified.

Our study also suggested that β-catenin might mediate the cooperative function of TGF-β signaling and Wnt signaling during the early stage of odontogenic differentiation. The interaction of TGF-β and Wnt signaling has been reported and explained in several developmental events, such as formation of the organizer and differentiation of chondrocytes (Nishita et al., 2000*;* Zhou et al., 2004). Several studies reported the importance of target gene co-regulation by TGF-β and Wnt signaling throughout development. In human marrow stromal cells, TGF-β synergize with Wnt signaling in the inhibition of adipocyte differentiation (Zhou et al., 2004). In the *Ocn-Cre; Ctnnb1^*fl/fl*^* mouse model that we generated previously, we and others discovered a critical role of β-catenin in root dentinogenesis (Kim et al., 2013*;* Zhang et al., 2013). However, how β-catenin is regulated during root odontoblast differentiation is largely unknown. The reduced β-catenin expression in *Ocn-Cre; Tgfbr2^*fl/fl*^; Wls^*fl/fl*^* differentiated root odontoblasts suggests the possible mechanism that the integrity of β-catenin is synergistically regulated by TGF-β and Wnt signaling, leading to undifferentiated odontoblasts and extremely thin root dentin. These data are consistent with the observations of other studies that TGF-β signaling interacts with Wnt signaling to activate the transcription of several genes via β-catenin-mediated signaling (Nishita et al., 2000*;* Zhou et al., 2004*;* Liu et al., 2006). TGF-β and Wnt signaling interacts physically by forming a complex composed of β-catenin, Lef1/Tcf-1 and Smad4 to bind to the *twin* (*Xtwn*) promoter region. This interaction directly and synergistically affects expression of the *twin* gene during formation of the organizer in *Xenopus* (Nishita et al., 2000). To test this hypothesis, future studies should constitutively stabilize β-catenin in mice to rescue the arrested root odontoblast differentiation in *Ocn-Cre; Tgfbr2^*fl/fl*^; Wls^*fl/fl*^* mice.

## Data Availability Statement

The original contributions presented in the study are included in the article/Supplementary Material, further inquiries can be directed to the corresponding author/s.

## Ethics Statement

The animal study was reviewed and approved by the Beijing Experimental Animal Regulation Board (SYXK/JING/2005/0031).

## Author Contributions

RZ contributed to conception and design, methodology, data acquisition, analysis, interpretation, and drafted the manuscript. JL, SY, and YL contributed to data acquisition and analysis and drafted the manuscript. QH contributed to data interpretation and analysis and drafted the manuscript. LZ contributed to data acquisition and drafted the manuscript. XY contributed to conception and design and critically revised the manuscript. GY contributed to conception and design, methodology, data acquisition, analysis, interpretation, and critically revised the manuscript. All authors gave final approval and agreed to be accountable for all aspects of the work.

## Conflict of Interest

The authors declare that the research was conducted in the absence of any commercial or financial relationships that could be construed as a potential conflict of interest.
