# Supplementary material for: Transforming Growth Factor-β Signaling Regulates Tooth Root Dentinogenesis by Cooperation With Wnt Signaling
Source: Front Cell Dev Biol. 2021 Jun 29;9:687099. doi: 10.3389/fcell.2021.687099 (PMC8277599; doi:10.3389/fcell.2021.687099)
Supplement: Supplementary file 1 [file Presentation_1.pdf]

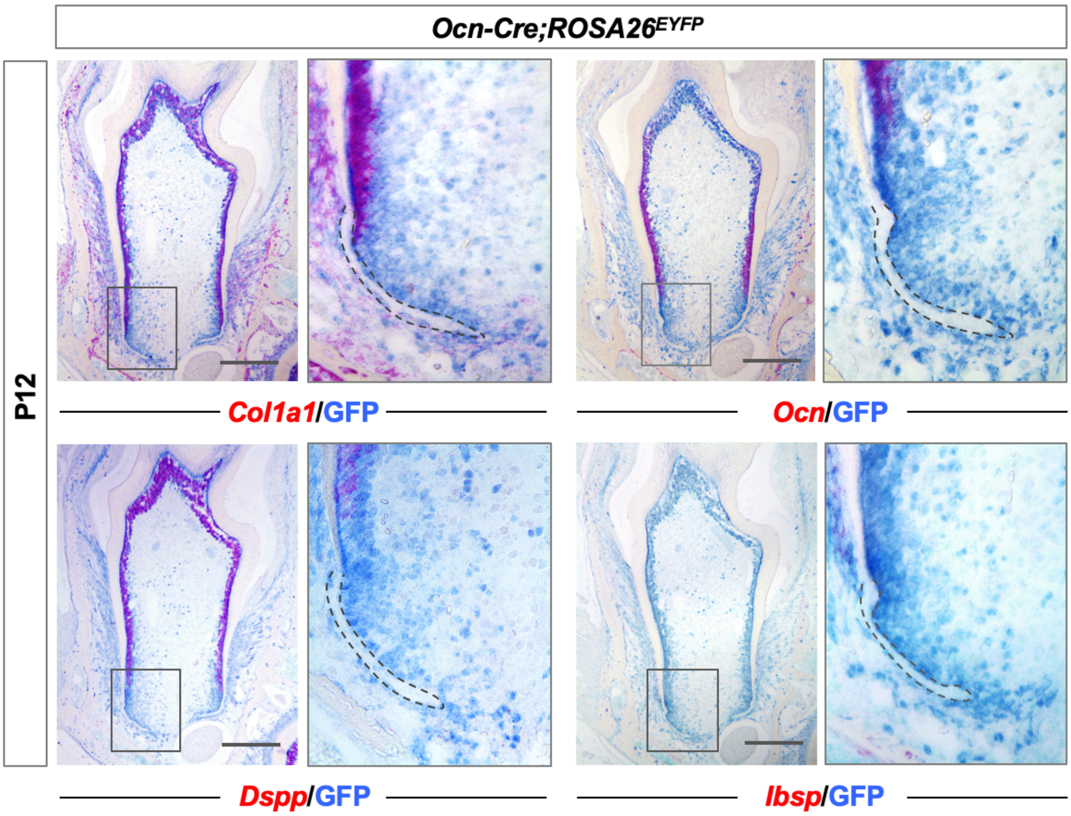

Supplementary Figure 1. *Ocn-Cre*-mediated gene recombination in developing teeth. n = 4 per genotype. Double staining of EYFP by immunostaining (blue, using anti-GFP antibody) and transcripts of *Col1a1, Ocn*, *Dspp*, *Ibsp* by *in situ* hybridization (fuchsia) in *Ocn-Cre;ROSA26*^{EYFP} molars at P12. Scale bars: 100 µm.

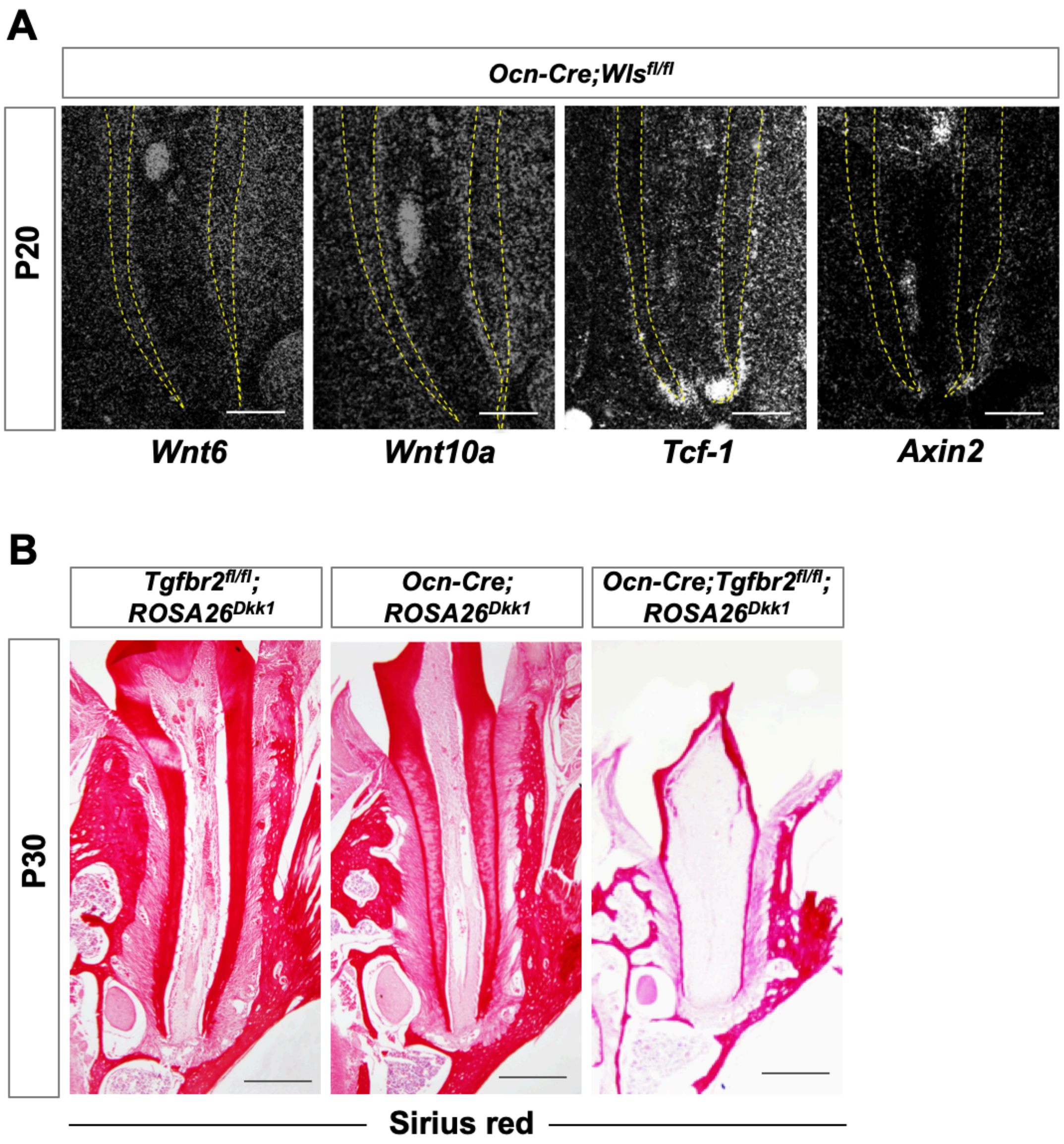

Supplementary Figure 2. Expression of Wnt related genes in *Ocn-Cre;Wls*$^{fl/fl}$ mice and decreased dentin thickness in *Ocn-Cre;Tgfbr2*$^{fl/fl}$*;ROSA26*^{Dkk1} mice. n = 4 mice/per genotype. (A) *In situ* hybridization of Wnt related gene expression in *Ocn-Cre;Wls*$^{fl/fl}$ mice. (B) Sirius red staining sections reveal shorter molar roots and thinner root dentin layer in molars of *Ocn-Cre;Tgfbr2*$^{fl/fl}$*;ROSA26*^{Dkk1} mice compared to *Tgfbr2*$^{fl/fl}$*;ROSA26*^{Dkk1} mice and *Ocn-Cre;ROSA26*^{Dkk1} mice at P30. Scale bars: 50 μm (A), 100 μm (B).
